# Information needs for making clinical recommendations about potential drug-drug interactions: a synthesis of literature review and interviews

**DOI:** 10.1186/s12911-017-0419-3

**Published:** 2017-02-22

**Authors:** Katrina M. Romagnoli, Scott D. Nelson, Lisa Hines, Philip Empey, Richard D. Boyce, Harry Hochheiser

**Affiliations:** 1 Department of Biomedical Informatics, University of Pittsburgh, Pittsburgh, PA USA; 2 Department of Biomedical Informatics, Vanderbilt University Medical Center, Nashville, TN USA; 3 Pharmacy Quality Alliance, Springfield, VA USA; 4 School of Pharmacy and Therapeutics, University of Pittsburgh, Pittsburgh, PA USA; 5 Intelligent Systems Program, University of Pittsburgh, Pittsburgh, PA USA

**Keywords:** Decision support systems, Clinical, Drug interactions, Drug information services, Information needs, Clinical recommendations

## Abstract

**Background:**

Drug information compendia and drug-drug interaction information databases are critical resources for clinicians and pharmacists working to avoid adverse events due to exposure to potential drug-drug interactions (PDDIs). Our goal is to develop information models, annotated data, and search tools that will facilitate the interpretation of PDDI information. To better understand the information needs and work practices of specialists who search and synthesize PDDI evidence for drug information resources, we conducted an inquiry that combined a thematic analysis of published literature with unstructured interviews.

**Methods:**

Starting from an initial set of relevant articles, we developed search terms and conducted a literature search. Two reviewers conducted a thematic analysis of included articles. Unstructured interviews with drug information experts were conducted and similarly coded. Information needs, work processes, and indicators of potential strengths and weaknesses of information systems were identified.

**Results:**

Review of 92 papers and 10 interviews identified 56 categories of information needs related to the interpretation of PDDI information including drug and interaction information; study design; evidence including clinical details, quality and content of reports, and consequences; and potential recommendations. We also identified strengths/weaknesses of PDDI information systems.

**Conclusions:**

We identified the kinds of information that might be most effective for summarizing PDDIs. The drug information experts we interviewed had differing goals, suggesting a need for detailed information models and flexible presentations. Several information needs not discussed in previous work were identified, including temporal overlaps in drug administration, biological plausibility of interactions, and assessment of the quality and content of reports. Richly structured depictions of PDDI information may help drug information experts more effectively interpret data and develop recommendations. Effective information models and system designs will be needed to maximize the utility of this information.

**Electronic supplementary material:**

The online version of this article (doi:10.1186/s12911-017-0419-3) contains supplementary material, which is available to authorized users.

## Background

A potential drug-drug interaction (PDDI) occurs when a patient is exposed to two or more drugs that are known to interact. Robust and accurate information regarding the potential adverse impacts of co-administration of drugs is critical for reducing the health impacts and costs of adverse events. The importance of this information is demonstrated by the existence of numerous print and online resources that summarize PDDI information, including institutional prescribing guidelines. Unfortunately, these resources also illustrate the challenges faced in interpreting PDDI information. An analysis of 14 publicly available resources identified wide variation in content, with the overlap between of interacting drug pairs between any two sources at generally less than 50% [1]. Several studies have found similarly substantial disagreement in the content of commercial sources of PDDI information [2–4].

The disagreements between resources that provide PDDI information are likely the result of the complexity of the task and the variability of the goals. Relevant details are distributed across multiple sources, including published literature, regulatory documents, drug labels, and case reports, each of which must be evaluated for credibility on its own merits and with respect to other, potentially conflicting, reports. Differences in goals may also account for discrepancies. One reviewer aiming to comprehensively identify all PDDIs may include information that might be ignored by a different reviewer who is interested only in likely, serious, and actionable interactions. Although some standardized practices for evaluating PDDI information have been developed [5, 6], concerns about the potential negative impact of methodological variations have led to calls for increased rigor [3].

Our broader goal – and the specific focus of this paper – is to develop information models and tools that will provide drug-drug interaction experts with clear and usable views of structured PDDI evidence, thus facilitating interpretation and hopefully increasing the quality and utility of PDDI information that reaches clinicians and patients. We are currently developing a semantic model of PDDI evidence suitable for creating structured descriptions of relevant reports, results, attribution of claims, and similar details necessary for assessing the clinical implications of PDDI descriptions [1, 7–10]. To populate these models, we are also developing annotation tools suitable for extracting these details from case reports, clinical trials, regulatory documents, product labels, and tertiary literature [11, 12]. A pilot study of the feasibility of engaging non-expert users to conduct such annotations suggests that these tools might be useful for crowd-sourcing the extraction of relevant PDDI information [13].

Although useful for bringing needed structure to PDDI information, these models and tools will not be accepted by users if they do not appropriately meet important information needs and support well-established practices. The work of reviewing and interpreting PDDI evidence is conducted by a relatively small and understudied group of drug information specialists with advanced PDDI knowledge. Here, we examine prior literature and interview responses from these experts to develop information needs and workflow models suitable for informing the design of informatics tools in support of PDDI information interpretation. The combination of data models, annotation tools, and an inexpensive approach to annotation presents the possibility of normalizing PDDI information on a large scale, turning complex unstructured textual reports into structured knowledge bases suitable for interrogation by experts involved in creation of compendia, guidelines, and similar resources. These tools will also be of interest to regulatory agencies responsible for providing guidance to industry regarding reporting of PDDI information [14–17].

### Objective

To identify factors influencing the interpretation and use of information necessary for making clinical recommendations about PDDIs, and to use the experience of practitioners to provide context for these factors. The key questions are:

1. What knowledge and factors influence drug decision making regarding PDDIs?
2. What is the workflow for seeking and synthesizing PDDI information during tasks such as clinical consultations for medication therapy decisions and guideline development?
3. What barriers do drug information experts report as obstacles to satisfying their information needs during PDDI information synthesis?

## Methods

### Overview

We conducted a literature search of the knowledge and factors that influence medication decision-making regarding PDDIs and workflows for seeking and synthesizing PDDI information; structured interviews with four potential users of an evidence assessment tool; and unstructured interviews with six drug information experts. Papers from the literature and interview transcripts were used to generate lists of information needs and a workflow model describing the process of reviewing PDDI information.

### Literature search

Two PharmD pharmacists (LH and PE) and a biomedical informatician (RDB), each with extensive knowledge of drug information and drug interactions, suggested potentially relevant articles based on their knowledge and experience. Of the articles discussed by the group, seventeen (Additional file 1: Appendix A) were selected as “seed” articles for developing a search strategy. MeSH terms and other keywords from these papers were used to develop MEDLINE and EMBASE queries (Additional file 1: Appendix B). After removing duplicates, two authors (KMR and RDB) independently screened the identified titles and abstracts to verify relevance to the project objectives (above). Disagreements were resolved by discussion.

**Fig. 1:**
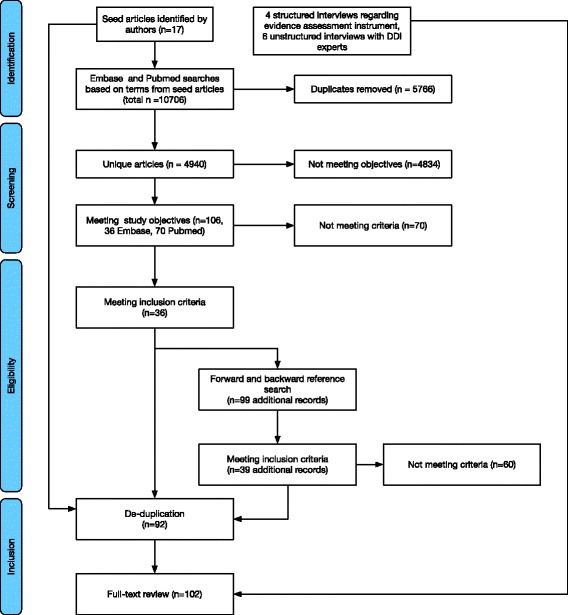
Literature search flow diagram

These papers were then screened for inclusion/exclusion using criteria focused on information needs for synthesis and evaluation of PDDI information, as opposed to resources dealing with PDDIs from the perspective of epidemiology or clinical pharmacology (Additional file 1: Appendix C). Automated methods for extracting PDDI information, such as through natural language processing were excluded. Regulatory guidelines describing requirements for discussion of drug-drug interaction information in drug labels were also excluded, as these documents act as specifications for product labeling and are not focused on information needs [14–17]. Papers citing and cited by the included papers were identified and subjected to a full-text review based on a broader set of inclusion/exclusion criteria by two of the authors (KMR and HH), resulting in a final set of papers for review. A summary of the search workflow can be found in Fig. 1.

Two authors (KMR and SN) conducted an open-coding qualitative analysis of the included articles to create a hierarchical coding scheme [18, 19] identifying information needs and factors relevant to making clinical recommendations about PDDIs. Initial categories for coding were derived from the study objectives described above, from Friedman & Wyatt’s nine types of evaluation studies in informatics [20], and from Goldschmidt’s workflow for information synthesis [21]. This codebook was expanded and refined from this initial list through multiple rounds of coding. Specifically, a subset of articles was reviewed and codes from the codebook were associated with specific spans in the document as needed. Inter-rater reliability for this coding was measured at the document level using Cohen’s Kappa [22]. Disagreements were discussed and resolved by consensus, and the process was repeated. After a total of 10 iterations, the coding team discussed the codes and developed a consensus categorization of the codes into higher-level categories and hierarchies. Inter-rater reliability was evaluated for this categorization using Cohen’s Kappa, pooled across all of the codes [23]. All coding activities were conducted using the QSR NVivo® coding software. The codebook used in this analysis is available from the authors upon request.

### Expert interviews

We conducted unstructured interviews with six experts involved in the synthesis of PDDI information. Participants included clinical pharmacists, drug information compendia editors, and academic drug information specialists who had previously participated in a conference series focusing on improving PDDI information [3]. Questions addressed respondents’ workflows, information needs, and information synthesis processes when conducting clinical consultations on drug-drug interactions and guideline development. When possible, respondents were asked to illustrate key aspects of their workflows and interpretive practices. Interviews were conducted by phone and web conference, during which the conversations were recorded via audio recording and screen capture of participants’ computers, when possible. We also reviewed transcripts of four interviews discussing a tool to help standardize the assessment of evidence for the existence of a drug-drug interaction [3]. All interviews were approved as exempt by the University of Pittsburgh Institutional Review Board.

Interview recordings were transcribed verbatim. As one interview was not recorded due to technical difficulties, interviewer notes were used in place of recording transcripts. Texts were coded using the same codebook used for the analysis of the literature search, with new codes added as needed to code concepts not found in the literature review. Graphical workflow models were developed to illustrate participants’ workflows, information seeking behavior, criteria for evaluating information, decision-making criteria, and other relevant aspects of the process of synthesizing PDDI information.

### Model generation

Codes from the literature search were reviewed to extract categories of information needs and specific types of information representing each of the categories. Information needs identified through analysis of expert interviews and through the construction of graphical workflows were added to the categories identified from the literature search, through a consensus process involving two of the authors (KMR and HH). These needs were added to the qualitative codebook and interviews were recoded to account for these new codes. The resulting codes were reviewed by two authors (KMR and HH) to identify recurring information needs. Individual workflows were condensed into a consensus model, informed by Goldschmidt’s model of information synthesis [21].

## Results

### Literature review and interview informants

Our original set of 17 articles (Additional file 1: Appendix A) led to another 36 articles identified during the database search. Reference searches for these articles led to the identification of an additional 39 articles. After de-duplication and the inclusion of seven additional gray-literature articles identified by the authors, the final set included 92 sources (Fig. 1) which were coded along with the four interviews regarding the proposed PDDI evidence assessment instrument and six interviews with compendia editors [3]. The full list of papers analyzed is available in Additional file 1: Appendix D. A subset of slightly over 20% of the papers (22 out of 92, 23.9%) was double-coded.

Inter-rater reliability of the final coding was substantial (pooled kappa = 0.66).

### PDDI information needs

Analysis of the literature review and interviews led to the identification of 56 primary information needs, summarized and classified into four categories (Table 1, full details including links to specific literature references and identification of needs identified in interviews given in Additional file 1: Appendix D). *Drug and interaction information* includes basic information known about the drugs involved in any study and their hypothetical roles in potential drug-drug interactions. *Study design* information needs reflect the criteria used by experts to assess the scientific applicability and validity of clinical and experimental studies, addressing issues such as experimental design, characteristics of participants, and relevance of doses. Examples of pertinent patient characteristics included clinical profiles of the participants, as healthy individuals might not be appropriate proxies for individuals experiencing symptoms treated by the medication. Similarly, studies involving dosages larger than typically prescribed might be considered uninformative. *Evidence* information needs were further divided into three subcategories: *quality and content of report* describing aspects of result reporting that might impact validity judgments*, clinical factors* describing the context of the interactions, and *consequences* enumerating possible adverse outcomes. *Recommendations,* the final category of information need, describes clinical actions that might be used to avoid potentially adverse effects.

**Table 1:** Information needs for interpreting potential drug-drug interaction reports, with indications of sources identifying those needs (literature and/or interviews) and counts of the number of sources mentioning each need

Category	Source	Subcategory	Information needs
Drug and Interaction Information	Literature and Interviews		Mechanisms of action (45 sources); pharmacokinetics (34); temporal overlap in administration of interacting drugs (27); pharmacodynamics (22); frequency of co-administration (16); category (drug class/related drugs) (14); biological plausibility of interaction (14); interaction role (object/precipitant) (4);
Study Design (randomly controlled trials)	Interviews		Dosage (5); participant characteristics (4); number of participants (3); controls (2); sample size calculation (1);
Evidence	Literature and Interviews	Quality and content of report	Drug Interaction Probability Scale [38] scores (10); differentiation between statistical and clinical significance (3); statistical characterization of results (3); inclusion of result magnitude (3); lack of evidence of interactions (2); thoroughness of new drug application (1); inclusion of human (non-animal) data as more credible (1); omissions of important details (1); Number of cases (1)
		Patient Factors	Clinical status (50); demographics (28); medication history (10); allergies (7); body weight (7); lifestyle (6); compliance (4); inter-patient variability (4); number of prescribers/pharmacies (2); length of hospital stay (1); payer status (1)
		Clinical	Dose (28); risk factors for consequences (21); clinical context (15); mitigating factors (11);
		Seriousness	Clinical importance (25); likelihood of irreversible morbidity (17); likelihood of mortality (14); likelihood of prescriber action (2)
		Adverse effects	Frequency of adverse events (numeric and/or estimated) (10); toxicity (6); reversibility of adverse effects (2); alteration of therapeutic effect (2);
Recommendations	Literature and Interviews		Monitor (33); dose adjustment (25); change medication (20); contraindication (20); discontinue or temporarily hold medication (13); modify administration (11); patient education (10); alternative therapy (10); strength of recommendation (9); continue treatment (7); when to start/stop management (2); seek medical attention (1); cost-effectiveness of recommendation (1)

### Expert interviews

Discussions with PDDI information experts suggested a range of personalized and informal practices. PDDI information review generally began either with a request for information from colleagues or clients, or from new papers or reports describing potential interactions. Informants relied on a variety of resources to identify relevant information, including PubMed (the most frequently mentioned resource), Google searches, Medwatch event reports, existing compendia, and new drug announcements. Although one respondent indicated that direct inquiries to manufacturers were occasionally useful, there was little enthusiasm across all respondents for the use of product labels.

Interviewees discussed a number of heuristics for evaluating both trials and case reports. Patient characteristics, dosages of potentially interacting drugs, temporal overlap of the drugs, and general plausibility of the PDDI were among the characteristics used to evaluate the relevance and importance of both clinical trials and case reports. Respondents discussed the use of specific criteria for evaluating both the design and the reporting of randomized controlled trials, describing the use of appropriate sample size calculations, controls, and participants and detailed reporting of specific effect sizes and statistical reports (area under the curve changes, p-values, confidence values) ﻿as indicative of high-quality design. There was a clear agreement that descriptions of theoretically-possible interactions were much less compelling than observed interactions. Along these lines, one respondent, who focused on Food and Drug Administration new drug announcements, discussed placing higher weight on reports involving human data, as opposed to results only observed in animal studies.

Respondents used a combination of heuristic and subjective processes to synthesize interpretations, attempting to derive conclusions from multiple, potentially conflicting reports. Considerations of the likely impact of the interaction, in terms of prevalence and seriousness, were frequently cited, with some respondents indicating that a single, sufficiently serious report might be sufficient to reach the conclusion that a PDDI was serious enough to merit concern. Although interviewees shared an interest in using sound science and considering all of the available information, differences in intended uses of recommendations led to different preferences in terms of tradeoffs between false positives and false negatives. Specifically, one respondent who edited a drug information compendium indicated a desire to be comprehensive, including all interactions supported by high levels of credible evidence, while another interviewee who made recommendations for drug alerting systems was much more concerned about the potential negative implications of false positives, instead preferring only interactions with high likelihoods of serious consequences.

**Fig. 2:**
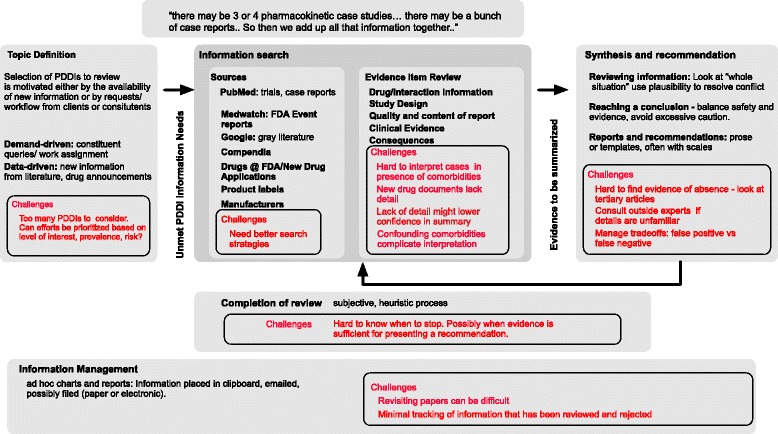
Potential Drug-Drug Interaction information evaluation and synthesis workflow

The PDDI review processes described by the interviewees were personal and informal. Most reviews started with online searches for literature (primarily through PubMed) and other resources, Evaluation of results, selections of resources, and interpretations was conducted informally, with no dedicated support from information tools such as reference management software or databases. Although some interviewees described filing of papers (either electronic copies or printed copies), no respondents mentioned any systematic effort to track which sources were reviewed in the course of evaluating any particular PDDI. One interviewee indicated that it was easier to conduct searches from scratch than to take the time to catalog which sources had been reviewed. The determination of when sufficient information had been gathered to make a recommendation was described as generally heuristic and subjective, focused on gathering sufficient data for a recommendation, as opposed to systematic investigation of all available evidence. Contents of recommendations included free text; structured reports providing recommendations on a scale of severity ranging from “no interaction” to “never prescribe together”; and summaries using structured templates described in the literature [6]. A graphical summary of the PDDI evidence evaluation workflow is given in Fig. 2.

## Discussion

Our review of 92 papers and interviews with ten domain experts provides a preliminary catalog of the types of information that are needed to interpret the importance of a PDDI. Although basic information on the drugs involved and the nature of the potential interaction are obvious starting points, more detail is needed to interpret the clinical importance of a PDDI. Contextual information, such as patient-specific risk factors and the likelihood of occurrence of a serious adverse event, is critical to guide useful recommendations. The quality, strength, relevance, and source of the evidence about an interaction matter as well: evidence from large clinical trials involving patients may be given more credence than in vitro experiments or data from animal studies.

Although many similar themes were identified both in the reviewed literature and in our interviews, the latter provided valuable insight into the process of interpreting evidence for PDDIs (Fig. 2). Experts described personalized and heuristic evidence review processes designed to assess the validity of the study design and the reported results. For example, reports of specific numeric magnitudes of effect sizes were described as being more trustworthy and convincing than comparable reports describing effects in qualitative terms (e.g. as “substantial increase”). Resulting reports or recommendations were also fairly subjective in content, although one respondent did discuss using a structured template to describe results [6].

Reducing the substantial impact of adverse events caused by drug-drug interactions is in many ways an information challenge. Clear, accurate, and useful PDDI information can help clinicians choose medication regimens that reduce the risk of adverse events. Although drug information specialists work diligently to interpret and synthesize relevant information for use by clinicians, the conversion of available evidence into guidance is a cognitively complex process, involving the extraction of information from research publications, event reports, product labels, and other free-text sources; the evaluation of the relevance and credibility of that information; and the construction of informative summaries. The difficulties inherent in these processes are reflected in differences in coverage and content across various drug-drug interaction knowledge bases, whether or not they were developed for commercial purposes [1, 24–26]. Several research efforts have attempted to tackle the challenge of extracting and representing PDDI information through techniques including natural language processing and crowdsourcing [13, 27–32]. However, much less attention has been paid to the processes of evaluating this information and synthesizing evidence.

Concern over the quality of PDDI information is not a new finding from this study. Our results are consistent with findings from recent examinations of drug-drug interaction resources and information models. Our literature review and interviews identified all of the data elements identified in our earlier survey of 14 publicly available drug-drug interaction information resources [1]. More recently, Herrero-Zazo, et al. examined 15 conceptual models of drug-drug interactions, identifying a set of 19 concepts [33]. Although categorizations and terms differ, information needs identified in our survey include all of these 19 concepts. Our results in this paper included several elements not found in either of these earlier efforts, including temporal overlaps in drug administration, biological plausibility of interactions, and assessments of the quality and content of the reports.

In response to concerns over PDDI information quality, a workgroup convened in early 2013 developed a consensus set of recommendations for evaluation of PDDI evidence, including the development of DRIVE: the Drug Interaction eVidence Evaluation instrument [3]. Although the recommendations of the workgroup are generally consistent with the information needs identified in our study, gaps between the workflows discussed during our interviews and the systematic evaluation proposed by the workgroup are substantial. Our expert interviews clearly suggest that the process of synthesizing PDDI information is idiosyncratic and highly personalized, with individuals differing on approaches to information seeking and interpretation, as well as the importance they ascribed to different aspects of the information. The development of software tools for managing the PDDI evidence synthesis workflow as a systematic process (as intended by DRIVE), while also supporting significant variations in information management, is an important product subject of future research.

Our findings also offer some suggestions to regulatory agencies responsible for providing guidance on PDDI content in drug product labels. Consistent with our results, current US Food and Drug Abbreviation guidelines suggest the inclusion of recommendations for dose adjustments, discussion of mechanisms of interaction, details of changes in concentrations of drugs or metabolites, and description of the source of the results. US guidelines also suggest the inclusion of any interference with lab tests [14], a factor not identified in our study. The inclusion of details identified in our study, including discussion of specifics of relevant patient factors; temporal factors; study designs and related reports; and adverse event seriousness might make the PDDI content in drug labels more useful both for drug information specialists and for practicing clinicians. Several of these elements, including temporal factors and related recommendations; clear recommendations; details specific to subpopulations; and variability of the response are discussed in European labeling guidelines [16]. Information needs might also play a role in considerations about the format of drug product labels. As currently specified in US guidelines, PDDI information might be found in distributed across multiple label sections including one dedicated to drug interactions [14]. Consolidation of all of these details into the drug interactions section, as opposed to cross-referencing those other sections, might increase the utility of these labels.

### Limitations

There were two limitations to our literature review. First, as the search terms were identified based on the seed list of 17 papers, biases in this list may have influenced our searches and the resulting list of papers considered for inclusion. Second, as our literature review did not include a systematic search of gray literature or non-English publications, our synthesis of factors identified in the literature may be incomplete. Other potential limitations include the small number of interviews: inclusion of additional experts may have led to the identification of additional factors or processes, along with potentially clearer insight into the prevalence and importance of information needs and related factors. As most of the discussion in the interviews involved descriptions of workflows, rather than demonstration of work in context, interview contents and analysis may suffer from problems of recall and de-contextualization. Additional interviews involving observation of DDI evidence review *in situ* might provide additional insights [19, 34]. Although our analytic processes, including coding by multiple reviewers and consensus revisions to codebooks, should increase the generalizability of our interpretations, standard risks of biases of qualitative research apply.

## Conclusion

Improvements in the quality and utility of clinically-relevant descriptions of PDDIs have the potential to contribute to reductions in both adverse events and, through clinical decision support systems, related costs [35]. Identifying relevant PDDI information research literature and other sources is highly complex. As this process will require manual curation for the foreseeable future [13], greater understanding of the information needs associated with PDDI information synthesis will be necessary to inform the design and content of tools designed to help drug information specialists conduct systematic and rigorous reviews. Our thematic analysis of the literature and interviews with experts provide a detailed view of the breadth and depth of necessary information, and the workflows associated with the processes. These insights will inform our ongoing efforts to develop richer information models for structured representation of drug-drug interaction evidence [1, 8, 9, 36]. The combination of these enhanced models with improvements to the usability of drug-drug interaction decision support presents the possibility for significant improvements in drug safety [37].

## Additional file

Additional file 1:
**A**: A list of seed publications used to develop the literature search. **B**: The search strategy used in the information search. **C**: Inclusion/exclusion criteria used in the literature review. **D**: Full list of information needs identified in the Literature Review and Interviews. (DOCX 478 kb)

## Abbreviations

DRIVE: the Drug Interaction eVidence Evaluation
PDDI: Potential Drug-Drug Interaction
