# Supplementary material for: Information needs for making clinical recommendations about potential drug-drug interactions: a synthesis of literature review and interviews
Source: BMC Med Inform Decis Mak. 2017 Feb 22;17:21. doi: 10.1186/s12911-017-0419-3 (PMC5322613; doi:10.1186/s12911-017-0419-3)
Supplement: Additional file 1: — A: A list of seed publications used to develop the literature search. B: The search strategy used in the information search. C: Inclusion/exclusion criteria used in the literature review. D: Full list of information needs identified in the Literature Review and Interviews. (DOCX 478 kb) [file 12911_2017_419_MOESM1_ESM.docx]

**Appendix A: Seed Publications**

Albanese, N. P., & Rouse, M. J. (2010). Scope of contemporary pharmacy practice: roles, responsibilities, and functions of pharmacists and pharmacy technicians. *Journal of the American Pharmacists Association : JAPhA*, *50*(2), e35–69. Retrieved from http://www.ncbi.nlm.nih.gov/pubmed/20199947

Duke, J. D., & Bolchini, D. (2011). A successful model and visual design for creating context-aware drug-drug interaction alerts. *AMIA . Annual Symposium Proceedings / AMIA Symposium. AMIA Symposium*, *2011*, 339–48. Retrieved from <http://www.pubmedcentral.nih.gov/articlerender.fcgi?artid=3243201&tool=pmcentrez&rendertype=abstract>

Floor-Schreudering, A., Geerts, A. F. J., Aronson, J. K., Bouvy, M. L., Ferner, R. E., & De Smet, P. a G. M. (2014). Checklist for standardized reporting of drug-drug interaction management guidelines. *European Journal of Clinical Pharmacology*, *70*(3), 313–8. http://doi.org/10.1007/s00228-013-1612-7

Hines, L. E. (2010). *Deadly Errors of Commission: Principles of Clinically Important Drug-Drug Interactions: An Interactive Case-Based Approach CME*.

Kannampallil, T. G., Jones, L. K., Patel, V. L., Buchman, T. G., & Franklin, A. (2014). Comparing the information seeking strategies of residents, nurse practitioners, and physician assistants in critical care settings. *Journal of the American Medical Informatics Association : JAMIA*, 1–8. http://doi.org/10.1136/amiajnl-2013-002615

Mutebi, A., Warholak, T. L., Hines, L. E., Plummer, R., & Malone, D. C. (2013). Assessing patients’ information needs regarding drug-drug interactions. *Journal of the American Pharmacists Association*, *53*(1), 39–45. http://doi.org/10.1331/JAPhA.2013.12038

Riedmann, D., Jung, M., Hackl, W. O., & Ammenwerth, E. (2011). How to improve the delivery of medication alerts within computerized physician order entry systems: an international Delphi study. *Journal of the American Medical Informatics Association*, *18*(6), 760–766. http://doi.org/10.1136/amiajnl-2010-000006

Russ, A. L., Saleem, J. J., Justice, C. F., Woodward-Hagg, H., Woodbridge, P. A., & Doebbeling, B. N. (2010). Electronic health information in use: Characteristics that support employee workflow and patient care. *Health Informatics Journal*, *16*(4), 287–305. http://doi.org/10.1177/1460458210365981

Russ, A. L., Zillich, A. J., McManus, M. S., Doebbeling, B. N., & Saleem, J. J. (2009). A human factors investigation of medication alerts: barriers to prescriber decision-making and clinical workflow. *AMIA ... Annual Symposium Proceedings / AMIA Symposium. AMIA Symposium*, *2009*, 548–52. Retrieved from http://www.pubmedcentral.nih.gov/articlerender.fcgi?artid=2815493&tool=pmcentrez&rendertype=abstract

Russ, A. L., Zillich, A. J., McManus, M. S., Doebbeling, B. N., & Saleem, J. J. (2012). Prescribers’ interactions with medication alerts at the point of prescribing: A multi-method, in situ investigation of the human-computer interaction. *International Journal of Medical Informatics*, *81*(4), 232–243. http://doi.org/S1386-5056(12)00013-5 [pii]\r10.1016/j.ijmedinf.2012.01.002

Schlaifer, M., & Rouse, M. J. (2015, September 14). Scope of Contemporary Pharmacy Practice: Roles, Responsibilities, and Functions of Pharmacists and Pharmacy Technicians. *Journal of Managed Care Pharmacy*. Academy of Managed Care Pharmacy. Retrieved from http://www.jmcp.org/doi/abs/10.18553/jmcp.2010.16.7.507

Seidling, H. M., Klein, U., Schaier, M., Czock, D., Theile, D., Pruszydlo, M. G., … Haefeli, W. E. (2014). What, if all alerts were specific - estimating the potential impact on drug interaction alert burden. *International Journal of Medical Informatics*, *83*(4), 285–91. <http://doi.org/10.1016/j.ijmedinf.2013.12.006>

Smithburger, P. L., Buckley, M. S., Bejian, S., Burenheide, K., & Kane-Gill, S. L. (2011). A critical evaluation of clinical decision support for the detection of drug drug interactions. *Expert Opinion on Drug Safety*, *10*(6), 871–882. <http://doi.org/10.1517/14740338.2011.583916>

Tang, D. H., Warholak, T. L., Hines, L. E., Hurwitz, J., Brown, M., Taylor, A. M., … Malone, D. C. (2014). Evaluation of Pharmacy and Therapeutic (P&T) Committee member knowledge, attitudes and ability regarding the use of comparative effectiveness research (CER) in health care decision-making. *Research in Social & Administrative Pharmacy : RSAP*, *10*(5), 768–80. <http://doi.org/10.1016/j.sapharm.2013.11.008>

Villa, L., Warholak, T. L., Hines, L. E., Taylor, A. M., Brown, M., Hurwitz, J., … Malone, D. C. (2013). Health Care Decision Makers’ Use of Comparative Effectiveness Research: Report from a Series of Focus Groups. *Journal of Managed Care Pharmacy*, *19*(9), 745–754. Retrieved from <Go to ISI>://000326430900003

Weideman, R. A., Bernstein, I. H., & McKinney, W. P. (1999). Pharmacist recognition of potential drug interactions. *Am J Health Syst Pharm*, *56*, 1524–1529. Retrieved from http://www.ncbi.nlm.nih.gov/entrez/query.fcgi?cmd=Retrieve&db=PubMed&dopt=Citation&list_uids=10478990

Zheng, K., Fear, K., Chaffee, B. W., Zimmerman, C. R., Karls, E. M., Gatwood, J. D., … Pearlman, M. D. (2011). Development and validation of a survey instrument for assessing prescribers’ perception of computerized drug-drug interaction alerts. *Journal of the American Medical Informatics Association*, *18*(Suppl 1), i51–i61. http://doi.org/10.1136/amiajnl-2010-000053

# Appendix B

**Search Strategy**

**PubMed**

((("Health Knowledge, Attitudes, Practice"[Mesh] OR "Attitude of Health Personnel"[Mesh] OR "Physician's Practice Patterns"[Mesh] OR "Decision Making"[Mesh] OR "Decision Making/drug effects"[Mesh] OR "Decision Making/methods"[Mesh] OR "Decision Making, Computer-Assisted"[Mesh] OR "Decision Making, Organizational"[Mesh] OR "Education, Pharmacy"[Mesh] OR "Education, Pharmacy/standards"[Mesh] OR "patient education as topic/methods"[Mesh] OR "Reminder systems"[Mesh] OR "Knowledge bases"[Mesh] OR "Drug Therapy, computer-assisted/methods"[Mesh] OR "Drug Therapy, Computer-Assisted"[Mesh]) AND

("Drug Interactions"[Mesh] OR "Drug Interactions/prevention and control"[Mesh] OR "Drug Interactions/drug effects"[Mesh] OR "Medication Errors"[Mesh] OR "Medication Errors/adverse effects"[Mesh] OR "Medication Errors/prevention and control"[Mesh] OR "Drug-Related Side Effects and Adverse Reactions"[Mesh] OR "Drug-Related Side Effects and Adverse Reactions/prevention and control"[Mesh]))

OR

("Attitude of Health Personnel"[Mesh] AND "Interviews as Topic"[Mesh] AND "Workflow"[Mesh]) NOT

("news"[Publication Type] OR "comment"[Publication Type] OR "editorial"[Publication Type] OR "newspaper article"[Publication Type]))

**2371 results (abstracts available)**

**Search translated to Embase:**  'drug information'/mj OR 'drug interaction'/mj OR 'drug surveillance program'/mj OR 'computerized provider order entry'/mj OR 'hospital information system'/mj OR 'decision support system'/mj OR 'computer assisted drug therapy'/mj OR 'health personnel attitude'/mj OR 'workflow'/mj OR 'professional knowledge'/mj OR 'pharmacy'/mj AND 'drug interaction'/de AND ('human'/de OR 'interview'/de OR 'qualitative research'/de OR 'questionnaire'/de) AND ('article'/it OR 'article in press'/it OR 'book'/it OR 'conference abstract'/it OR 'conference paper'/it OR 'review'/it) AND [embase]/lim

**8434 results**

**Embase search limited to adverse drug reaction or drug interaction subheadings:** 'drug information'/mj OR 'drug interaction'/mj OR 'drug surveillance program'/mj OR 'computerized provider order entry'/mj OR 'hospital information system'/mj OR 'decision support system'/mj OR 'computer assisted drug therapy'/mj OR 'health personnel attitude'/mj OR 'workflow'/mj OR 'professional knowledge'/mj OR 'pharmacy'/mj AND 'drug interaction'/de AND ('human'/de OR 'interview'/de OR 'qualitative research'/de OR 'questionnaire'/de) AND ('article'/it OR 'article in press'/it OR 'book'/it OR 'conference abstract'/it OR 'conference paper'/it OR 'review'/it) AND [embase]/lim AND ('adverse drug reaction':lnk OR 'drug interaction':lnk)

**1416 results**

# Appendix C Inclusion/Exclusion Criteria

Articles were included if they were studies examining:

- Factors that influence and support clinical decision-making regarding PDDIs
- Information needs and information seeking behavior of persons who synthesize PDDI information during tasks such as medication therapy management, consulting, drug information, and guideline development
- Clinicians’ knowledge of PDDIs and self-efficacy when encountering PDDIs
- Factors that influence the use and efficacy of PDDI alerts
- Drug safety/risk communication, prevention/identification of medications errors, and prevention/identification adverse drug events that are relevant to PDDIs
- Or consensus statements, qualitative studies including surveys, interviews, literature reviews, conference proceedings, and white papers

First round exclusion criteria and number of articles excluded:

| **Criterion** | **Number of Articles Excluded** |
| --- | --- |
| Epidemiologic studies examining prevalence and/or risks for drug-related problems (without specific mention of PDDI knowledge or databases) | 5 |
| Studies of the medication therapy process, polypharmacy, clinical decision support unrelated to PDDIs | 12 |
| Anything with Schedule I controlled substances, environmental toxins, herbal-drug and food-drug interactions, dietary-supplement - drug interaction, or alcohol-drug interaction | 4 |
| Studies targeting pharmaceutical scientists | 2 |
| Studies of text mining and natural language processing algorithms, even if applied to PDDIs | 0 |
| Lacks all of the following: drug interaction specificity, or examination of the factors that influence drug interactions, or the information needs or information seeking behavior of clinicians related to drug interactions. | 32 |
| Specific PDDI case studies, "dear doctor" articles, or letters to the editor because they are unlikely to add anything compared to the information-based studies | 8 |
| Not in English | 7 |
| **Total** | 70 |

Second round exclusion criteria and number of articles excluded:

| **Number of Articles Excluded** | **Number of Articles Excluded** |
| --- | --- |
| Epidemiologic studies examining prevalence and/or risks for drug-related problems  (without specific mention of DDI knowledge or databases) | 14 |
| Studies of the medication therapy process, polypharmacy, clinical decision support not specific to drug-drug interactions | 1 |
| Anything with Schedule I controlled substances, environmental toxins, herbal-drug and food-drug interactions, dietary-supplement - drug interaction, or alcohol-drug interaction | 0 |
| Studies targeting pharmaceutical scientists | 2 |
| Studies of text mining and natural language processing algorithms, even if applied to drug-drug interactions | 0 |
| Lacks all of the following: drug interaction specificity, or examination of the factors that influence drug interactions, or the information needs or information seeking behavior of clinicians related to drug interactions. | 4 |
| Specific DDI case studies, "dear doctor" articles, or letters to the editor because they are unlikely to add anything compared to the information-based studies | 3 |
| General, high-level discussions identifying DDI as a problem and framing issue in general terms | 11 |
| Compendia discordance analysis | 14 |
| prevalence study of DDI interactions | 1 |
| DDI Discussions not related to information use or needs | 1 |
| Compendium of DDI information | 2 |
| Evaluations of expert curated DDI lists without replicable details | 7 |
| Total | 60 |

**Appendix D Full List of Information Needs**

**Supplemental Data**

**Methods:** 92 papers from the primary and gray literature, four interviews regarding the usability of the DRIVE drug interaction evidence assessment tool, and six interviews with drug information compendia editors were analyzed to identify information needs and factors relevant to making clinical recommendations about potential drug-drug interaction. Emergent qualitative coding was just to generate a set of codes that was revised through a consensus process. After coding, codes were revised and re-categorized to provide an overall consensus of pertinent issues. These categorizations were further summarized to produce lists of information needs and indicators of success/failure for clinical information systems, as given in Figures 3 and 4 of the paper. Categories, related codes, and sources are given below.

**INFORMATION NEEDS**

| **Category** | **Subcategory** | **Code** | **Subcode/**  **Description** | **Source:** | | |
| --- | --- | --- | --- | --- | --- | --- |
|  |  |  |  | **DRIVE Interviews** | **DDI Expert Interviews** | **Literature** |
| Drug and Interaction Information |  | Category (drug class or related drugs) | Category (drug class/related drugs), drugs with related pharmacokinetic impacts/side effects |  | 2 | [1–12] |
|  |  | Pharmacodynamics |  |  | 1 | [3,6–8,11–27] |
|  |  | Mechanisms of action |  | 1 | 4 | [1–9,11,12,14–18,25–48] |
|  |  | Pharmacokinetics | Elimination, metabolism, pathways | 2 | 4 | [3,6–8,11,12,14–27,29,30,37,38,40,46,49,50] |
|  |  | Object Drug/Precipitant Drug |  |  | 1 | [7,18,21] |
|  |  | Frequency of co-administration |  |  |  | [2,6,7,12,14,16,21,27,28,36,38,39,51–54] |
|  |  | Biological Plausibility |  | ` | 2 | [7,8,10,11,15,21,33,34,36,39,47,55] |
|  | Timing | Temporal overlap in administration of interacting drugs | Interaction occurs upon discontinuation, interaction timing, time of interaction onset, time-dependent drug-drug interaction, timing or temporal separation of medications |  | 4 | [1,2,6–9,11,12,14,17,18,21–23,26,36,37,43,46,49,53,56,57] |
| Study Design (randomly controlled trials) |  | Number of participants |  |  | 3 |  |
|  |  | Controls |  |  | 2 |  |
|  |  | Dosage |  |  | 5 |  |
|  |  | sample size calculation |  |  | 1 |  |
|  |  | participant characteristics |  |  | 4 |  |
| Evidence | Quality and content of report | Differentiation between statistical and clinical significance |  |  | 3 |  |
|  |  | Thoroughness of new drug application |  |  | 1 |  |
|  |  | statistical characterization of results |  |  | 3 |  |
|  |  | inclusion of human (non-animal) data as more credible |  |  | 1 |  |
|  |  | inclusion of result magnitude |  |  | 3 |  |
|  |  | lack of evidence of interactions |  |  | 2 |  |
|  |  | omissions of important details references |  |  | 1 |  |
|  |  | DIPS scores |  | 2 | 5 | [11,18,29] |
|  |  | Number of cases |  |  | 1 |  |
|  | Patient Factors | Allergies |  |  |  | [12,22,44,58–61] |
|  |  | Body Weight |  |  |  | [7,12,22,33,37,44,62] |
|  |  | Clinical Status |  |  | 2 | [2,5,7,9–13,17–19,21–25,27,28,30,32,33,35–38,40,43,44,47–50,52,56,58,59,62–71] |
|  |  | Compliance |  |  |  | [18,32,62,72] |
|  |  | Demographics |  |  |  | [2,5,7,10–12,17,19–23,27,33,36–38,40,43,44,47,49,58,61,66,67,69,72] |
|  |  | Inter-patient variability |  |  | 1 | [7,19,67] |
|  |  | Length of hospital stay |  |  |  | [49] |
|  |  | Lifestyle |  |  |  | [2,5,7,12,20,36] |
|  |  | Medication history |  |  |  | [5,10,12,22,28,31,44,67,69,72] |
|  |  | Number of prescribers or pharmacies |  |  |  | [7,21] |
|  |  | Payer status |  |  |  | [49] |
|  | Clinical | Dose |  |  | 2 | [2,3,6,11,12,14–16,18–20,22–24,26,35–37,40,43,44,47,49,56,60,61] |
|  |  | Clinical Context |  |  | 1 | [1,2,9,11,15,27,28,32,34,43–45,73,74] |
|  |  | Modifying factors(including mitigating factors and risk factors) | Mitigating factors |  | 1 | [2,7,11,12,18,23,26,38,39,69] |
|  |  |  | Risk factors for consequences |  | 1 | [1,7,9,11,12,14,17,18,21–23,26,32,34,37,38,43,47,55,69] |
|  | Seriousness | Clinical importance |  |  |  | [1,2,6,7,9,11,12,17,21,24–26,28,33,34,42,47,49,51,53,56,62,75–77] |
|  |  | Likelihood of irreversible morbidity |  |  | 2 | [6,7,12,21–23,28,33,34,39,47,49,51,59,75] |
|  |  | Likelihood of mortality |  |  | 2 | [3,6,7,12,21,33,34,39,47,51,75,78] |
|  |  | Likelihood of prescriber action |  |  |  | [21,75] |
|  | Adverse Effects | Toxicity |  |  | 1 | [11,13,40,50,57] |
|  |  | Reversibility of adverse effects |  |  |  | [22,57] |
|  |  | Alteration of therapeutic effect |  |  |  | [11,15] |
|  | Consequences | Frequency of consequences | Estimated statement of frequency |  |  | [11,12,34,63] |
|  |  |  | Numerical statement of frequency |  |  | [11,12,16,34,45,63] |
| Recommendations |  | Change Medication |  |  | 1 | [6–9,11,14,15,17,19,28,33,38,39,43,56,57,72,79] |
|  |  | Monitoring |  |  |  | [1–4,6–9,11,12,15,17,19,26–28,33,34,36–39,43,49,55–57,62,69,72,78–80] |
|  |  | Modify Administration |  |  |  | [6,12,15,19,28,33,37,46,56,57,79] |
|  |  | Patient Education |  |  |  | [6,11,22,28,37,49,54,56,57,78] |
|  |  | Continue Treatment |  |  |  | [9,28,33,39,62,78,79] |
|  |  | Discontinue or temporarily hold medication |  |  |  | [1,7,9,12,18,28,33,39,43,56,57,78,79] |
|  |  | Contraindication |  |  |  | [3,6–9,12,17,22,23,33,37,39,40,56,62,63,70,72,79,80] |
|  |  | Alternative therapy |  |  |  | [2,9,11,14,15,22,37,42,57,67] |
|  |  | Dose Adjustment |  |  | 1 | [1–3,6–8,11,12,14,15,17,19,22,28,33,37–39,43,50,56,57,62,79] |
|  |  | Seek medical attention |  |  |  | [49] |
|  |  | When to start/stop management |  |  |  | [49] |
|  |  | Treatment plan |  |  |  | [22] |
|  |  | Strength of recommendation |  |  | 1 | [3,6,11,12,15,33,34,56] |
|  |  | Cost-effectiveness of recommendation |  |  |  | [34] |
| Papers included in analysis but not associated with any of the above summary codes | | | | | | [81–92] |

**REFERENCES**

An online version of this bibliography is available at at <https://www.mendeley.com/groups/8811551/drug-drug-interaction-information-needs-literature-review/>

1 Barrons R. Evaluation of personal digital assistant software for drug interactions. *Am J Health Syst Pharm* 2004;**61**:380–5.http://www.ncbi.nlm.nih.gov/pubmed/15011766

2 Bergk V, Gasse C, Rothenbacher D, *et al.* Drug interactions in primary care: impact of a new algorithm on risk determination. *Clin Pharmacol Ther* 2004;**76**:85–96. doi:10.1016/j.clpt.2004.02.009

3 Bergk V, Haefeli WE, Gasse C, *et al.* Information deficits in the summary of product characteristics preclude an optimal management of drug interactions: a comparison with evidence from the literature. *Eur J Clin Pharmacol* 2005;**61**:327–35. doi:10.1007/s00228-005-0943-4

4 Hines LE, Ceron-Cabrera D, Romero K, *et al.* Evaluation of warfarin drug interaction listings in US product information for warfarin and interacting drugs. *Clin Ther* 2011;**33**:36–45. doi:10.1016/j.clinthera.2011.01.021

5 Lucente FE. Computerized Database of Drug Interactions: A Paradigm for Resolving a Communication Gap in Otolaryngology. *Laryngoscope* 1985;**95**:1367–73.

6 Luna D. Analysis and Redesign of a Knowledge Database for a Drug-drug Interactions Alert System. In: *Medinfo 2007: Proceedings of the 12th World Congress on Health (Medical) Informatics; Building Sustainable Health Systems*. 2007. 885–9.

7 Mallet L, Spinewine A, Huang A. The challenge of managing drug interactions in elderly people. *Lancet* 2007;**370**:185–91. doi:10.1016/S0140-6736(07)61092-7

8 Murphy JE, Malone DC, Olson BM, *et al.* Development of computerized alerts with management strategies for 25 serious drug-drug interactions. *Am J Health Syst Pharm* 2009;**66**:38–44. doi:10.2146/ajhp070046

9 Reis AMM, Cassiani SHDB. Evaluation of three brands of drug interaction software for use in intensive care units. *Pharm World Sci* 2010;**32**:822–8. doi:10.1007/s11096-010-9445-2

10 Riedmann D, Jung M, Hackl WO, *et al.* Development of a context model to prioritize drug safety alerts in CPOE systems. *BMC Med Inform Decis Mak* 2011;**11**:35. doi:10.1186/1472-6947-11-35

11 Scheife RT, Hines LE, Boyce RD, *et al.* Consensus recommendations for systematic evaluation of drug-drug interaction evidence for clinical decision support. *Drug Saf* 2015;**38**:197–206.http://www.ncbi.nlm.nih.gov/pubmed/25556085 (accessed 24 Jan2016).

12 Tilson H, Hines LE, McEvoy G, *et al.* Recommendations for selecting drug-drug interactions for clinical decision support. *Am J Health Syst Pharm* 2016;**73**:576–85.http://www.ajhp.org/content/73/8/576.abstract

13 Albanese NP, Rouse MJ. Scope of contemporary pharmacy practice: roles, responsibilities, and functions of pharmacists and pharmacy technicians. *J Am Pharm Assoc (2003)* 2010;**50**:e35–69.http://www.ncbi.nlm.nih.gov/pubmed/20199947

14 Bergk V, Gasse C, Schnell R, *et al.* Requirements for a successful implementation of drug interaction information systems in general practice: results of a questionnaire survey in Germany. *Eur J Clin Pharmacol* 2004;**60**:595–602. doi:10.1007/s00228-004-0812-6

15 Böttiger Y, Laine K, Andersson ML, *et al.* SFINX-a drug-drug interaction database designed for clinical decision support systems. *Eur J Clin Pharmacol* 2009;**65**:627–33. doi:10.1007/s00228-008-0612-5

16 Chan A, Yap KY-L, Koh D, *et al.* Electronic database to detect drug-drug interactions between antidepressants and oral anticancer drugs from a cancer center in Singapore: implications to clinicians. *Pharmacoepidemiol Drug Saf* 2011;**20**:939–47.http://www.ncbi.nlm.nih.gov/pubmed/21732473

17 Hines LE. Deadly Errors of Commission: Principles of Clinically Important Drug-Drug Interactions: An Interactive Case-Based Approach CME. 2010.

18 Horn JR, Hansten PD, Chan L-N. Proposal for a new tool to evaluate drug interaction cases. *Ann Pharmacother* 2007;**41**:674–80. doi:10.1345/aph.1H423

19 Horn JR. Why Can’ t We Just Get Relevant Alerts ? Pharm. Times. 2012;1–5.http://www.pharmacytimes.com/publications/issue/2012/december2012/why-cant-we-just-get-relevant-alerts

20 Long CO, Ismeurt RL, White PA. Preventing Drug Interactions in the Home: A Five-Step Approach for Client Teaching. *Home Healthc Nurse* 1999;**17**:106–12.

21 Malone DC, Abarca J, Hansten PD, *et al.* Identification of serious drug-drug interactions: results of the partnership to prevent drug-drug interactions. *J Am Pharm Assoc (2003)* 2004;**44**:142–51.http://www.ncbi.nlm.nih.gov/pubmed/15098848

22 Rahmner PB, Eiermann B, Korkmaz S, *et al.* Physicians’ reported needs of drug information at point of care in Sweden. *Br J Clin Pharmacol* 2012;**73**:115–25. doi:10.1111/j.1365-2125.2011.04058.x

23 Seidling HM, Klein U, Schaier M, *et al.* What, if all alerts were specific - estimating the potential impact on drug interaction alert burden. *Int J Med Inform* 2014;**83**:285–91. doi:10.1016/j.ijmedinf.2013.12.006

24 Smithburger PL, Buckley MS, Bejian S, *et al.* A critical evaluation of clinical decision support for the detection of drugdrug interactions. *Expert Opin Drug Saf* 2011;**10**:871–82. doi:10.1517/14740338.2011.583916

25 Trujillo J. INSTRUCTIONAL DESIGN AND ASSESSMENT A Drug Interactions Elective Course. *Am J Pharm Ed* 2009;**73**:1–8.

26 Vonbach P, Dubied A, Krähenbühl S, *et al.* Evaluation of frequently used drug interaction screening programs. *Pharm World Sci* 2008;**30**:367–74. doi:10.1007/s11096-008-9191-x

27 Zorina OI, Haueis P, Greil W, *et al.* Comparative performance of two drug interaction screening programmes analysing a cross-sectional prescription dataset of 84,625 psychiatric inpatients. *Drug Saf* 2013;**36**:247–58. doi:10.1007/s40264-013-0027-9

28 Armstrong EP, Wang SM, Hines LE, *et al.* Evaluation of a drug-drug interaction: fax alert intervention program. *BMC Med Inform Decis Mak* 2013;**13**:32. doi:10.1186/1472-6947-13-32

29 Boyce R, Collins C, Horn J, *et al.* Computing with evidence. Part I: A drug-mechanism evidence taxonomy oriented toward confidence assignment. *J Biomed Inform* 2009;**42**:979–89. doi:10.1016/j.jbi.2009.05.001

30 Boyce R, Collins C, Horn J, *et al.* Computing with evidence Part II: An evidential approach to predicting metabolic drug-drug interactions. *J Biomed Inform* 2009;**42**:990–1003. doi:10.1016/j.jbi.2009.05.010

31 Driesen A, Simoens S, Laekeman G. Management of drug interactions with beta-blockers : continuing education has a short-term impact. 2006;**4**:143–50.

32 Duke JD, Bolchini D. A successful model and visual design for creating context-aware drug-drug interaction alerts. *AMIA Annu Symp Proc* 2011;**2011**:339–48.http://www.pubmedcentral.nih.gov/articlerender.fcgi?artid=3243201&tool=pmcentrez&rendertype=abstract

33 Far E, Curkovic I, Byrne K, *et al.* Validation of a transparent decision model to rate drug interactions. *BMC Pharmacol Toxicol* 2012;**13**:7. doi:10.1186/2050-6511-13-7

34 Floor-Schreudering A, Geerts AFJ, Aronson JK, *et al.* Checklist for standardized reporting of drug-drug interaction management guidelines. *Eur J Clin Pharmacol* 2014;**70**:313–8. doi:10.1007/s00228-013-1612-7

35 Georget S, Lederlin C, Manciaux MA, *et al.* Comparison of drug interaction data bases. *J Pharm Clin* 1997;**16**:36–44.http://www.jle.com/en/revues/jpc/e-docs/comparaison_de_banques_de_donnees_sur_les_interactions_medicamenteuses_160075/article.phtml?tab=texte

36 Hansten PD. ORCA: OpeRational CalssificAtion of drug interactions. *J Am Pharm Assoc* 2001;**41**:161–5.

37 Hansten PD. Drug interaction management. *Pharm World Sci* 2003;**25**:94–7. doi:10.1023/A:1024077018902

38 Haueis P, Greil W, Huber M, *et al.* Evaluation of drug interactions in a large sample of psychiatric inpatients: a data interface for mass analysis with clinical decision support software. *Clin Pharmacol Ther* 2011;**90**:588–96. doi:10.1038/clpt.2011.150

39 Horn JR, Hansten PD. Customizing clinical decision support to prevent excessive drug-drug interaction alerts. *Am J Health Syst Pharm* 2011;**68**:662–5. doi:10.2146/ajhp100465

40 Knollmann BC, Smyth BJ, Garnett CE, *et al.* Personal digital assistant-based drug reference software as tools to improve rational prescribing: benchmark criteria and performance. *Clin Pharmacol Ther* 2005;**78**:7–18. doi:10.1016/j.clpt.2005.03.007

41 Lam M V, McCart GM, Tsourounis C. An Assessment of Free, Online Drug-Drug Interaction Screening Programs (DSPs). *Hosp Pharm* 2003;**38**:662–8.

42 Lopez-Picazo JJ. Percepcion de los medicos de familia acerca de un programa de ayuda a la prescripcion para reducir las interacciones medicamentosas. *Farm Aten Primaria* 2012;**10**:27–31.

43 Payne TH, Hines LE, Chan RC, *et al.* Recommendations to improve the usability of drug-drug interaction clinical decision support alerts. *J Am Med Inform Assoc* 2015;**22**:1243–50.http://jamia.oxfordjournals.org/content/early/2015/03/30/jamia.ocv011.abstract (accessed 24 Jan2016).

44 Phansalkar S, Hoffman JM, Hurdle JF, *et al.* Understanding pharmacist decision making for adverse drug event (ADE) detection. *J Eval Clin Pract* 2009;**15**:266–75. doi:10.1111/j.1365-2753.2008.00992.x

45 Poirier TI, Giudici R. Evaluation of Drug Interaction Microcomputer Software: Comparative Study. *Hosp Pharm* 1991;**26**:30–3.

46 van der Sijs H, Lammers L, van den Tweel A, *et al.* Time-dependent Drug-Drug Interaction Alerts in Care Provider Order Entry: Software May Inhibit Medication Error Reductions. *J Am Med Informatics Assoc* 2009;**16**:864–8. doi:10.1197/jamia.M2810

47 Van Roon EN, Flikweert S, Le Comte M, *et al.* Clinical relevance of drug-drug interactions: A structured assessment procedure. *Drug Saf* 2005;**28**:1131–9. doi:10.2165/00002018-200528120-00007

48 Weideman RA, Bernstein IH, McKinney WP. Pharmacist recognition of potential drug interactions. *Am J Heal Syst Pharm* 1999;**56**:1524–9.http://www.ncbi.nlm.nih.gov/entrez/query.fcgi?cmd=Retrieve&db=PubMed&dopt=Citation&list_uids=10478990

49 Mitchiner JC, Korzeniewski SJ, Betten D, *et al.* Evaluation of the B-SAFE campaign to reduce clinically significant warfarin-drug interactions among fee-for-service Medicare beneficiaries. *Am J Med Qual* 2012;**27**:518–23. doi:10.1177/1062860612438706

50 Seidling HM, Storch CH, Bertsche T, *et al.* Successful strategy to improve the specificity of electronic statin – drug interaction alerts. *Eur J Clin Phamacol* 2009;**11**:1149–57. doi:10.1007/s00228-009-0704-x

51 Abarca J, Malone DC, Armstrong EP, *et al.* Concordance of severity ratings provided in four drug interaction compendia. *J Am Pharm Assoc* 2004;**44**:136–41. doi:10.1331/154434504773062582

52 Abarca J, Colon LR, Wang VS, *et al.* Evaluation of the performance of drug-drug interaction screening software in community and hospital pharmacies. *J Manag Care Pharm* 2006;**12**:383–9.

53 Andersson ML, Böttiger Y, Lindh JD, *et al.* Impact of the drug-drug interaction database SFINX on prevalence of potentially serious drug-drug interactions in primary health care. *Eur J Clin Pharmacol* 2013;**69**:565–71. doi:10.1007/s00228-012-1338-y

54 Russ AL, Zillich AJ, McManus MS, *et al.* Prescribers’ interactions with medication alerts at the point of prescribing: A multi-method, in situ investigation of the human-computer interaction. *Int J Med Inform* 2012;**81**:232–43. doi:S1386-5056(12)00013-5 [pii]\r10.1016/j.ijmedinf.2012.01.002 [doi]

55 van der Sijs H, Aarts J, van Gelder T, *et al.* Turning off frequently overridden drug alerts: limited opportunities for doing it safely. *J Am Med Inform Assoc* 2008;**15**:439–48. doi:10.1197/jamia.M2311

56 Mille F, Schwartz C, Brion F, *et al.* Analysis of overridden alerts in a drug-drug interaction detection system. *Int J Qual Heal Care* 2008;**20**:400–5. doi:10.1093/intqhc/mzn038

57 Mutebi A, Warholak TL, Hines LE, *et al.* Assessing patients’ information needs regarding drug-drug interactions. *J Am Pharm Assoc* 2013;**53**:39–45. doi:10.1331/JAPhA.2013.12038

58 Böttiger Y, Eiermann B. Knowledge Databases for Rational Drug Therapy: Develompent, Usage, and Effects. In: *Basic & Clinical Pharmacology & Toxicology*. 2011. 48–54. doi:10.1111/j.1742-7843.2011.00721.x

59 Jankel CA, Martin BC. Evaluation of six computerized drug interaction screening programs. *Am J Hosp Pharm* 1992;**49**:1430–5.http://www.ncbi.nlm.nih.gov/entrez/query.fcgi?cmd=Retrieve&db=PubMed&dopt=Citation&list_uids=1529984

60 Lapane KL, Waring ME, Schneider KL, *et al.* A mixed method study of the merits of e-prescribing drug alerts in primary care. *J Gen Intern Med* 2008;**23**:442–6. doi:10.1007/s11606-008-0505-4

61 Poirier TI, Giudici RA. Evaluation of drug interaction microcomputer software: Medicom Micro Plus. *Hosp Pharm* 1989;**24**:273–7.http://www.mendeley.com/research/evaluation-drug-interaction-microcomputer-software-medicom-micro-plus/ (accessed 14 Jan2016).

62 Yeh ML, Chang YJ, Wang PY, *et al.* Physicians’ responses to computerized drug-drug interaction alerts for outpatients. *Comput Methods Programs Biomed* 2013;**111**:17–25. doi:10.1016/j.cmpb.2013.02.006

63 Duke JD, Li X, Grannis SJ. Data visualization speeds review of potential adverse drug events in patients on multiple medications. *J Biomed Inform* 2010;**43**:326–31. doi:10.1016/j.jbi.2009.12.001

64 Duke JD, Li X, Dexter P. Adherence to drug-drug interaction alerts in high-risk patients: a trial of context-enhanced alerting. *J Am Med Inform Assoc* 2013;**20**:494–8. doi:10.1136/amiajnl-2012-001073

65 Hines LE, Murphy JE, Grizzle AJ, *et al.* Critical issues associated with drug-drug interactions: highlights of a multistakeholder conference. *Am J Health Syst Pharm* 2011;**68**:941–6.http://www.ncbi.nlm.nih.gov/pubmed/21546646

66 Ko Y, Malone DC, D’Agostino J V, *et al.* Potential Determinants of Prescribers’ Drug-Drug Interaction Knowledge. *Res Soc Adm Pharm* 2008;**4**:355–66. doi:10.1016/j.sapharm.2007.10.004

67 Lopez-Picazo JJ, Ruiz JC, Sanchez JF, *et al.* A randomized trial of the effectiveness and efficiency of interventions to reduce potential drug interactions in primary care. *Am J Med Qual* 2011;**26**:145–53.http://ajm.sagepub.com/content/26/2/145

68 Mulherin DP, Zimmerman CR, Chaffee BW. National standards for computerized prescriber order entry and clinical decision support: The case of drug interactions. *Am J Heal Pharm* 2013;**70**:59–64. doi:10.2146/ajhp120217

69 Riedmann D, Jung M, Hackl WO, *et al.* How to improve the delivery of medication alerts within computerized physician order entry systems: an international Delphi study. *J Am Med Informatics Assoc* 2011;**18**:760–6. doi:10.1136/amiajnl-2010-000006

70 Shah VS, Weber RJ, Nahata MC. Contradictions in contraindications for drug-drug interactions. *Ann Pharmacother* 2011;**45**:409–11. doi:10.1345/aph.1P792

71 Woosley RL, Romero K. Assessing cardiovascular drug safety for clinical decision-making. *Nat Rev Cardiol* 2013;**10**:330–7. doi:10.1038/nrcardio.2013.57

72 Koplan KE, Brush AD, Packer MS, *et al.* ‘Stealth’ Alerts to Improve Warfarin Monitoring When Initiating Interacting Medications. *J Gen Intern Med* 2012;**27**:1–8. doi:10.1007/s11606-012-2137-y

73 Askari M. Relevance of drug-drug interaction in the ICU - perceptions of intensivists and pharmacists. *Stud Health Technol Inform* 2012;**180**:716–20.

74 Cornu P, Steurbaut S, Soštarić S, *et al.* Performance of a clinical decision support system and of clinical pharmacists in preventing drug-drug interactions on a geriatric ward. *Int J Clin Pharm* 2014;**36**:519–25. doi:10.1007/s11096-014-9925-x

75 Smithburger PL, Kane-Gill SL, Benedict NJ, *et al.* Grading the severity of drug-drug interactions in the intensive care unit: a comparison between clinician assessment and proprietary database severity rankings. *Ann Pharmacother* 2010;**44**:1718–24. doi:10.1345/aph.1P377

76 Spina JR, Glassman PA, Belperio P, *et al.* Clinical relevance of automated drug alerts from the perspective of medical providers. *Am J Med Qual* 2005;**20**:7–14. doi:10.1177/1062860604273777

77 Warholak TL, Hines LE, Saverno KR, *et al.* Assessment tool for pharmacy drug-drug interaction software. *J Am Pharm Assoc (2003)* 2011;**51**:418–24. doi:10.1331/JAPhA.2011.10054

78 Armstrong EP, Wang SM, Hines LE, *et al.* Prescriber perceptions of a near real-time fax alert program for potential drug-drug interactions. *J Manag care Spec Pharm* 2014;**20**:494–500a.http://www.ncbi.nlm.nih.gov/pubmed/24761821

79 Morrell J. Receptivity of Physicians in a Teaching Hospital to a Computerized Drug Interaction Monitoring and Reporting System. *Med Care* 1977;**XV**:68–78.

80 Glassman P a, Belperio P, Simon B, *et al.* Exposure to automated drug alerts over time: effects on clinicians’ knowledge and perceptions. *Med Care* 2006;**44**:250–6. doi:10.1097/01.mlr.0000199849.08389.91

81 Glassman P a, Belperio P, Simon B, *et al.* Exposure to automated drug alerts over time: effects on clinicians’ knowledge and perceptions. *Med Care* 2006;**44**:250–6. doi:10.1097/01.mlr.0000199849.08389.91

82 Hazlet TK, Lee TA, Hansten PD, *et al.* Performance of community pharmacy drug interaction software. *J Am Pharm Assoc* 2001;:200–4.

83 Kannampallil TG, Jones LK, Patel VL, *et al.* Comparing the information seeking strategies of residents, nurse practitioners, and physician assistants in critical care settings. *J Am Med Inform Assoc* 2014;:1–8. doi:10.1136/amiajnl-2013-002615

84 Lipman AG, Devenport JK, Page BC. Analysis of information provided by clinical pharmacists. *Am J Hosp Pharm* 1982;**39**:71–3.

85 Russ AL, Zillich AJ, McManus MS, *et al.* A human factors investigation of medication alerts: barriers to prescriber decision-making and clinical workflow. *AMIA Annu Symp Proc* 2009;**2009**:548–52.http://www.pubmedcentral.nih.gov/articlerender.fcgi?artid=2815493&tool=pmcentrez&rendertype=abstract

86 Russ AL, Saleem JJ, Justice CF, *et al.* Electronic health information in use: Characteristics that support employee workflow and patient care. *Health Informatics J* 2010;**16**:287–305. doi:10.1177/1460458210365981

87 Schlaifer M, Rouse MJ. Scope of Contemporary Pharmacy Practice: Roles, Responsibilities, and Functions of Pharmacists and Pharmacy Technicians. J. Manag. Care Pharm. 2015.http://www.jmcp.org/doi/abs/10.18553/jmcp.2010.16.7.507

88 Tang DH, Warholak TL, Hines LE, *et al.* Evaluation of Pharmacy and Therapeutic (P&T) Committee member knowledge, attitudes and ability regarding the use of comparative effectiveness research (CER) in health care decision-making. *Res Social Adm Pharm* 2014;**10**:768–80. doi:10.1016/j.sapharm.2013.11.008

89 Villa L, Warholak TL, Hines LE, *et al.* Health Care Decision Makers’ Use of Comparative Effectiveness Research: Report from a Series of Focus Groups. *J Manag Care Pharm* 2013;**19**:745–54.<Go to ISI>://000326430900003

90 Zachariah M, Phansalkar S, Seidling HM, *et al.* Development and preliminary evidence for the validity of an instrument assessing implementation of human-factors principles in medication-related decision-support systems--I-MeDeSA. *J Am Med Inform Assoc* 2011;**18 Suppl 1**:i62–72. doi:10.1136/amiajnl-2011-000362

91 Zheng K, Fear K, Chaffee BW, *et al.* Development and validation of a survey instrument for assessing prescribers’ perception of computerized drug-drug interaction alerts. *J Am Med Informatics Assoc* 2011;**18**:i51–61. doi:10.1136/amiajnl-2010-000053

92 Zhu X, Cimino JJ. Clinicians’ evaluation of computer-assisted medication summarization of electronic medical records. *Comput Biol Med* 2014;**59**:221–31. doi:10.1016/j.compbiomed.2013.12.006
